# Reverse shoulder arthroplasty in obstetric brachial plexus injury: our experience with shoulder motion analysis

**DOI:** 10.1186/s10195-023-00736-0

**Published:** 2023-11-10

**Authors:** Giuseppe Porcellini, Marco Montemagno, Chiara Manzini, Gabriele Fiumana, Andrea Giorgini, Gianmario Micheloni, Luigi Tarallo

**Affiliations:** 1 Orthopedic and Traumatology Department, University of Modena and Reggio Emilia, Modena, Italy; 2 Department of General Surgery and Medical Surgical Specialties, Section of Orthopaedics and Traumatology, University Hospital Policlinico-San Marco, University of Catania, Catania, Italy; 3 Shoulder Team S.R.L., Forlì, Italy

**Keywords:** Obstetric brachial plexus palsy, Erb palsy, Reverse shoulder arthroplasty, Shoulder motion analysis

## Abstract

**Background:**

Obstetric brachial plexus injury (OBPI) is a weakening or paralysis of the upper arm caused by brachial plexus injury followed by a muscle paralysis with severe repercussions on the movement of the shoulder joint following a progressive glenohumeral joint deformity.

This case series analyzes the clinical and radiological outcomes of reverse total shoulder arthroplasty (RSA) in OBPI patients with a follow-up of 2 years.

**Materials and methods:**

OBPI patients with secondary end-stage glenohumeral arthritis were enrolled in the study and they were treated with RSA. Patient demographics and clinical outcomes [Range of Motion (ROM), Visual Analog Scale (VAS), Oxford Shoulder Score (OSS)] were evaluated. A novel Shoulder motion analysis was carried out to investigate specific movement patterns of scapulothoracic movements in these patients. This study is a prospective cohort study.

**Results:**

Four Patients (M: *F* = 1:3) were enrolled in the study, the mean age was 49.3 years (+ 2.75), the mean OSS (Oxford Shoulder Score) decreased from 48.8 (± 2.5) preoperatively to 18.30 (± 2.78), the mean VAS (Visual Analog Scale) decreased from 7.25 (± 0.5) to 1.7 (± 0.3) in the follow up (∆% relative pain reduction:− 76.5%), Shoulder ROM obtained an improvement (*p* < 0.05) except for abduction and external rotation. The average follow-up time was 26.3 months (+− 4.5). Shoulder motion analysis showed a complete loss of the scapular tilting above 90 degrees of flexion compared to the typical one of standard RSA with a pattern shifted towards scapular retraction (engaging trapezius and rhomboid muscles) to compensate the loss of the posterior tilting.

**Conclusions:**

RSA in OBPI patients demonstrated a significant improvement of pain symptoms and a moderate improvement in daily activities, anyway with a more appreciable quality of life over time even if the marked hypotrophy especially of the posterior shoulder muscles showed some limits in maintaining suspension of the upper limb and a minor external rotation, with an internal rotation attitude during the movements.

*Level of evidence*: Level IV, Case series.

## Introduction

### Background

OBPI or Erb’s palsy is a weakening or paralysis of the upper arm caused by brachial plexus injury (stretch, rupture, or avulsion), followed by a muscle paralysis (contracture in adduction), with severe repercussions on the movement of the shoulder joint (internal rotation) and progressive glenohumeral joint deformity (retroverted and dysplastic glenoid, humeral head epiphysiolisys, down-sloping acromion, elongated and vertical coracoid).

The damage entity is related to the extension (C5-C6 are generally involved, sometimes in combination with C7 [1]) and the severity of the nerve damage.

The radicular injury, more often C5-C7 usually post-ganglionic, leads to muscle weakness in abduction, involving deltoid and supraspinatus, and external rotation, due to infraspinatus deficiency; therefore the shoulder appears adducted and internally rotated, with a consequent dysplasia and dislocation of the humeral head over time [2].

The primary cause of nerve injury lies in the abnormal mobilization of the baby’s head, during childbirth not properly performed; however, also with a cesarean section brachial plexus injury may occur [3].

The right upper limb is the most often involved since the left occipital anterior vertex presentation is the more frequent [1].

Patients with OBPI may compensate for limited glenohumeral motion through their scapulothoracic articulation [4].

The literature reports various data regarding the incidence of OBPI all over the world. It varies from 0.3 to 4 per 1000 live births [4]; between 1988 and 1997, at the Academic Medical Center in Amsterdam, the incidence of OBPI was 4.6 per 1000 [5].

Muscle strength recovery seems to occur from 76.2 to 90% of patients, although a residual contracture of the shoulder is frequently observed [5].

These patients generally envelop a degenerative disease of the shoulder at a younger age than in the general population [6]; actually, no data are known about the evolution of cases towards symptomatic osteoarthritis, probably due to their low number or to the difficulty of shoulder arthroplasty surgery.

The therapy mainly consists of conservative treatment and, when chronic and uncontrolled painful end-stage osteoarthritis occurs, shoulder arthroplasty is strongly suggested to restore a center of rotation for the glenohumeral joint, improving pain and returning a better quality of life [7]. When a glenoid erosion is present, the gold standard surgical treatment is represented by an RSA and custom-made glenoid implant, followed by a careful postoperative rehabilitation [8], focused on strengthening the anterior part of the deltoid and pectoralis major to increase arm flexion and the posterior part of the deltoid in abduction to increase external rotation [9, 10].

RSA consists of three main components: the baseplate, the glenosphere, and the humeral socket. It provides joint stabilization and pain resolution; furthermore, the lateralization of the center of rotation allows the deltoid to increase its strength during elevation and abduction, avoiding cuff deficiency-related limitations.

Because of the abnormal anatomic condition characterized by internal rotation contractures and subscapularis shortening, in OBPI patients some authors [4] prefer to leave the subscapularis unrepaired to avoid stiffness and limitation in the external rotation.

To focus attention on the needs and expectations of the patient is mandatory to outline the most suitable treatment case by case; moreover, any previous therapeutic or surgical treatment must be evaluated, to obtain the best outcome and minimum complications.

**Fig. 1:**
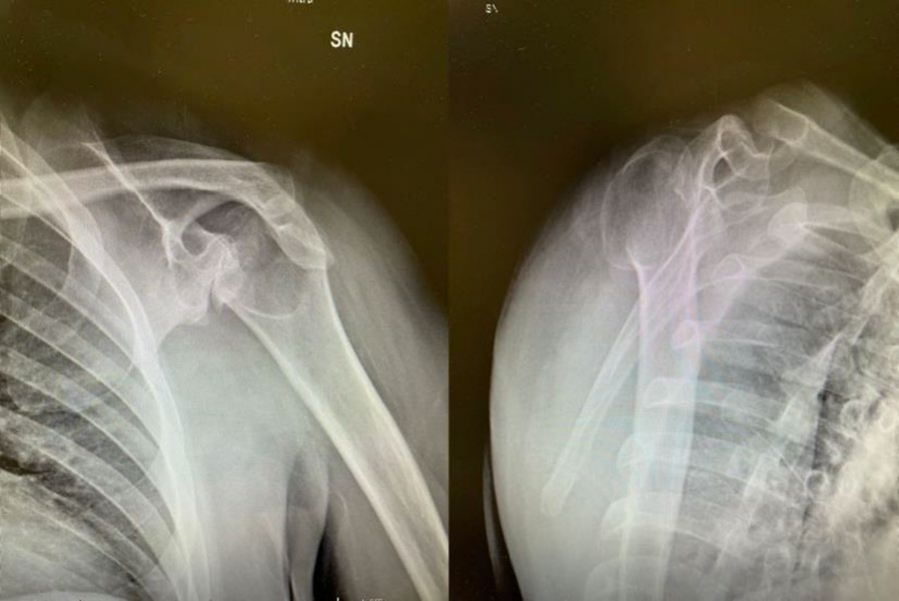
Antero-posterior and “Scapular-Y” Xrays views of OBPI shoulder in this study

**Fig. 2:**
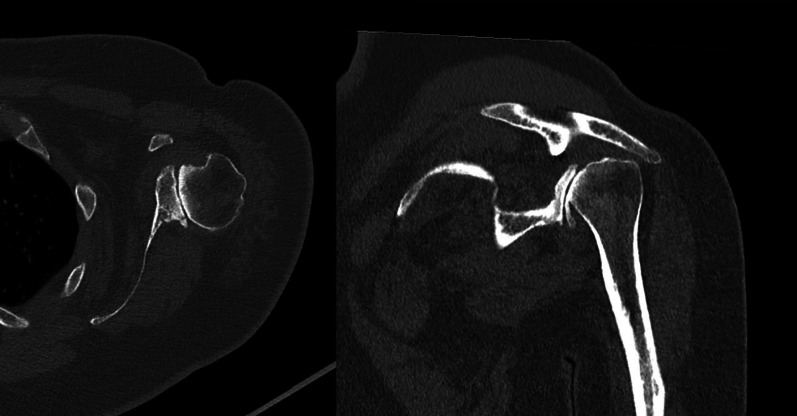
Axial and Sagittal CT scan views showing retroversion of glenoid

**Fig. 3:**
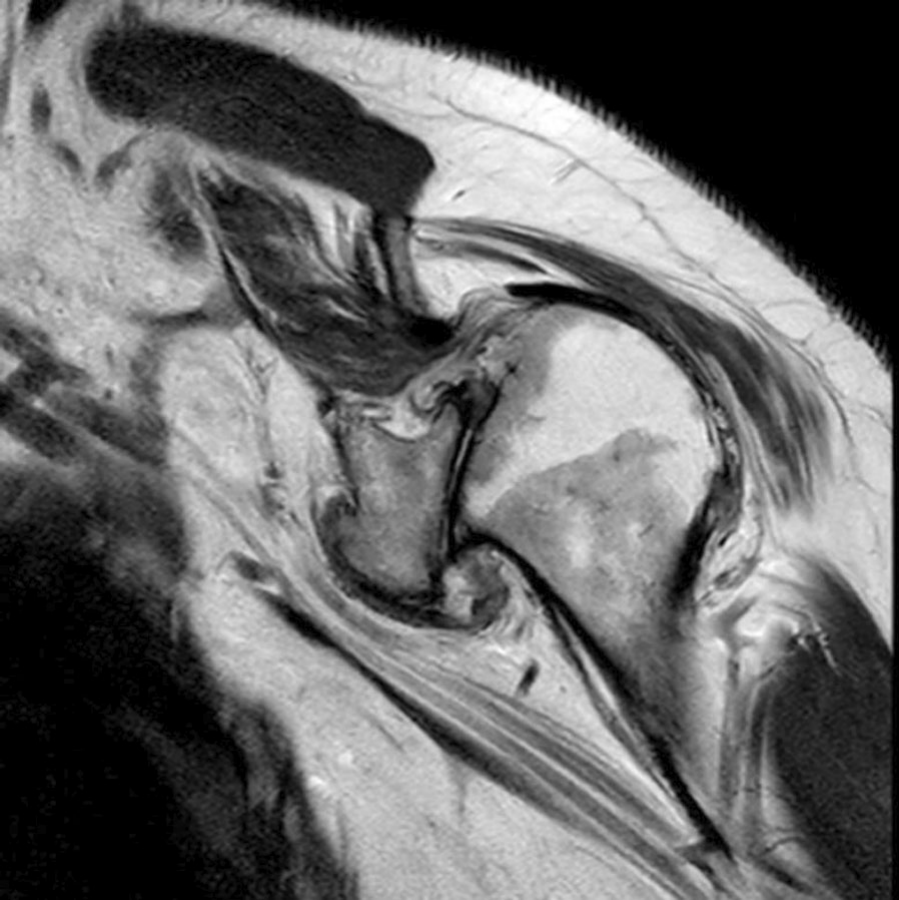
T2W sagittal plane MRI showing massive rotator cuff tear with abnormal arthritic erosion of humeral head

First of all, the surgical approach should require a physical examination of the neurovascular district and cervical spine; the shoulder range of motion, strength, and muscle function of the deltoid must be tested [11]. A careful radiologic evaluation (Fig. 1) of the preoperative state completes patient assessment by performing a 3D-CT (3D—Computer tomography) (Fig. 2)for the study of bone structures and an MRI (Magnetic Resonance Imaging) to evaluate rotator cuff integrity (Fig. 3).

The deltoid muscle is the key to RSA functioning but many authors [12, 13] demonstrated that the contribution of scapular rhythm to shoulder elevation increases significantly in the RSA shoulders.

Shoulder motion analysis is a new technical tool to accurately analyze the scapulothoracic joint during the movement of the upper arm. Its usefulness in the field of shoulder prostheses lies in understanding the movements in the three planes of the scapula, thus they can be divided into upper and lower tilting, ante- retro-positioning, and intra- extra- rotation. In literature [14] it is explained that the RSA motion pattern in normal subjects shows a more extensive range of motion in two different planes (scapular tilt and upward rotation), with an evident trend in the range of 30–90 degrees. This means that Patients with a shoulder prosthesis have a greater scapular motion compensation compared to the healthy side. As far as we know, no previous work on RSA in OBPI patients’ scapular kinematics has been performed.

### Objectives

Shoulder arthroplasty in OBPI is technically demanding because of the severe deformity, which is often characterized by glenoid medialization and retroversion and consequently the high risk of prosthesis dislocation because of the imbalance of the soft tissues [15, 16]. The aim of this study is to investigate the clinical outcomes of RSA in OBPI patients and to relate a specific motion pattern with shoulder motion analysis to understand better the scapulo-toracic compensation of these patients.

## Materials and methods

Between 2020 and 2021, all patients affected by OBPI with end-stage glenohumeral osteoarthritis were enrolled in this prospective study and treated with RSA replacement surgery at our Institute. Inclusion criteria: Patients affected by GH arthritis secondary to OBPI requiring RSA treated with Ascend Flex (Stryker, Kalamazoo, Michigan, USA) humeral component prostheses. The exclusion criteria were: age < 18 years, revision, or failed shoulder arthroplasty. Informed consent was applied. Basic preliminary patient information such as age at surgery, gender, affected side, dominant side, glenoid type (Walch classification), and glenoid version were collected.

The outcome variables considered were: Visual Analog Scale (VAS), Oxford Shoulder Score (OSS), and active ROM (Range of Motion in external rotation, internal rotation, abduction, flexion). The indication for RSA was uncontrolled pain (VAS > 5) and function impairment in end-stage osteoarthritis, after previous non-surgical therapies failure.

Preoperative X-rays, CT scans, and MRIs were obtained for each patient in the study. CT scans showed, for all our patients, a morphological feature of the glenoid referable to stage C, according to Walch classification [17, 18].

All patients underwent postoperative radiographs and serial clinical and radiological follow-up evaluations. They were carefully informed about the possible risks and complications of this kind of surgery.

RSA surgery was performed in all patients using a deltopectoral approach, using standard antibiotic prophylaxis according to allergies. Patients were hospitalized for 2 days after surgery.

Pre and post-operative OSS were submitted for all patients, consisting of 12 items, each with 5 potential answers. A mark between 1 (best/fewest symptoms) and 5 (worst/most severe) was assessed corresponding to the patient’s symptoms. The combined total gives a minimum score of 12 and a maximum of 60. A higher score implies a greater degree of disability [19].

All patients were evaluated at three months from surgery, for post-operative scapular mobility using Showmotion 3D kinematic tracking system analysis (NCS lab srl, Modena, Italy). The evaluation was performed on three Cartesian axes, related to the movements of the humerus flexion–extension, using seven detection sensors (wireless miniaturized inertial measurement units) placed on the wrist, the humerus, the spine of the scapula and the sternal manubrium. The anterior and posterior scapular tilting (transverse axes), the scapular protraction and retraction (vertical axes), and the mediolateral rotation (sagittal axis) has been evaluated by the system and compared to the lateral healthy side.

Through active ROM, the first motion required was anterior flexion, until the patient’s maximum available range without pain. The second motion was an abduction in the scapular plane to the patient’s maximum available range without pain. The third motion was internal and external rotation with the elbow adducted to the thorax. Each movement was repeated six times, starting from the healthy side. This tracking system is a reproducible and easy method for the assessment of scapular angular kinematics in healthy adults as described by literature [20].

Data were collected using mean and standard deviation, and for continuous variables, the *T*-student test was used to assess statistical significance of the results.

## Results

Four patients were enrolled in the study: they were 3 females and 1 male, with a mean age of 49.3 years (+− 2.75), and presented painful end-stage shoulder osteoarthritis according to most used classifications in literature [17, 21, 22]. They were all affected on the left side by OBPI (Table 1). None of them underwent previous surgery.

**Table 1:** OBPI patient’s pre-operative characteristics

	Patient 1	Patient 2	Patient 3	Patient 4
Age at surgery	46	51	52	48
Sex	Female	Female	Female	Male
Affected side	Left	Left	Left	Left
Dominant Arm	Right	Right	Left	Right
Glenoid Type (Walch classification)	B2	C	B2	B2
Glenoid Version	22	46	37	31

The average follow-up time for clinical evaluation was 26.3 months (+− 4.5). From the date of surgery.

Preoperative CT scans confirmed the posterior dislocation of the shoulder and dysplasia and retroversion of the glenoid surface.

Clinical evaluation before surgery could document an adducted and internally rotated shoulder with a very limited abduction due to muscle weakness, involving subscapularis and supraspinatus, and external rotation, for infraspinatus deficiency.

The OSS in post-surgery follow-up showed moderate improvement in activities of daily living but great in shoulder pain; the mean score decreased from 48.76 (± 2.5) preoperatively to 18.30 (± 2.78) (p < 0.05) in the follow-up (∆% relative pain reduction: -76.55%). Pain relief showed a higher score among the OSS items, VAS decreased from 7.25 (± 0.5) to 1.7 (± 0.3) (p < 0.05), according to the experience of other Authors [4, 7], who observed an improvement of pain but not in function.

**Fig. 4:**
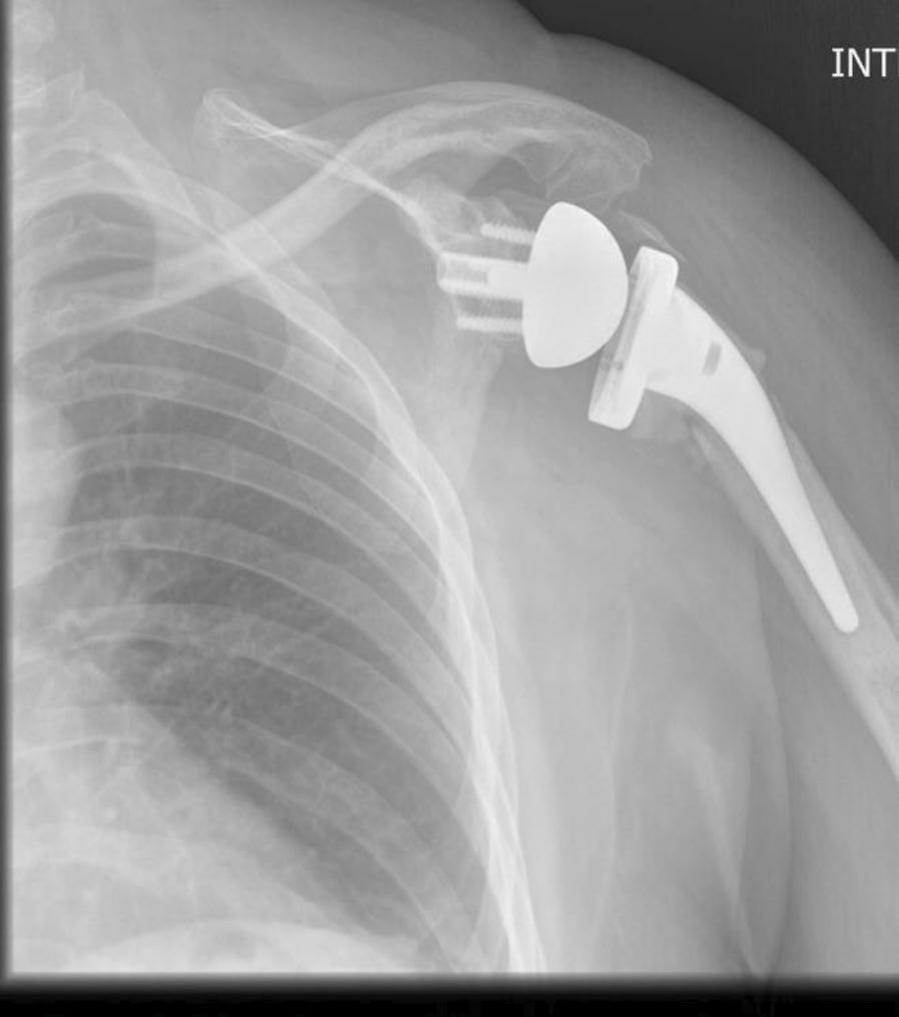
Postoperative Antero- posterior x rays of RSA in OBPI patient n.2

X-rays findings after surgery showed the correct placement of the prostheses (Fig. 4).

The active internal rotation and the active forward abduction movements were substantially superimposable pre-and post-operatively, while the flexion and external rotation movement showed a moderate improvement [active flexion: from 91.25° (± 6.3) to 122.55°(± 7.5); active external rotation: from 11.25° (± 6.3) to 17.5° (+ − 2.8)]. Regarding daily activities, patients noticed some improvement in dressing and driving a car but not, for example, in combing their hair, because of the inability to maintain the strength required for overhead function. The outcome results are shown in the table below (Table 2).

**Table 2:** Clinical results of RSA in OBPI in this study

	PRE	POST	*p*
OSS	48.76 (± 2.5)	18.30 (± 2.78)	< 0.05
VAS	7.25 (± 0.5)	1.7 (± 0.3)	< 0.05
Active ER	11.25 (± 6.3)	17.5° (+ − 2.8)	0.07
Active IR	71.25° (± 6.3)	76.25° (± 4.8)	0.09
Active FL	91.25° (± 6.3)	122.55° (± 7.5)	0.015
Active AB	78.7° (± 17.5)	98.75° (± 12.5)	0.06

Statistical significance is indicated with the *T*-student test

*PRE* pre-4 operative, *POST* post-operative, *ER* external rotation, *IR* internal rotation, *FL* Flexion, *AB* abduction

**Fig. 5:**
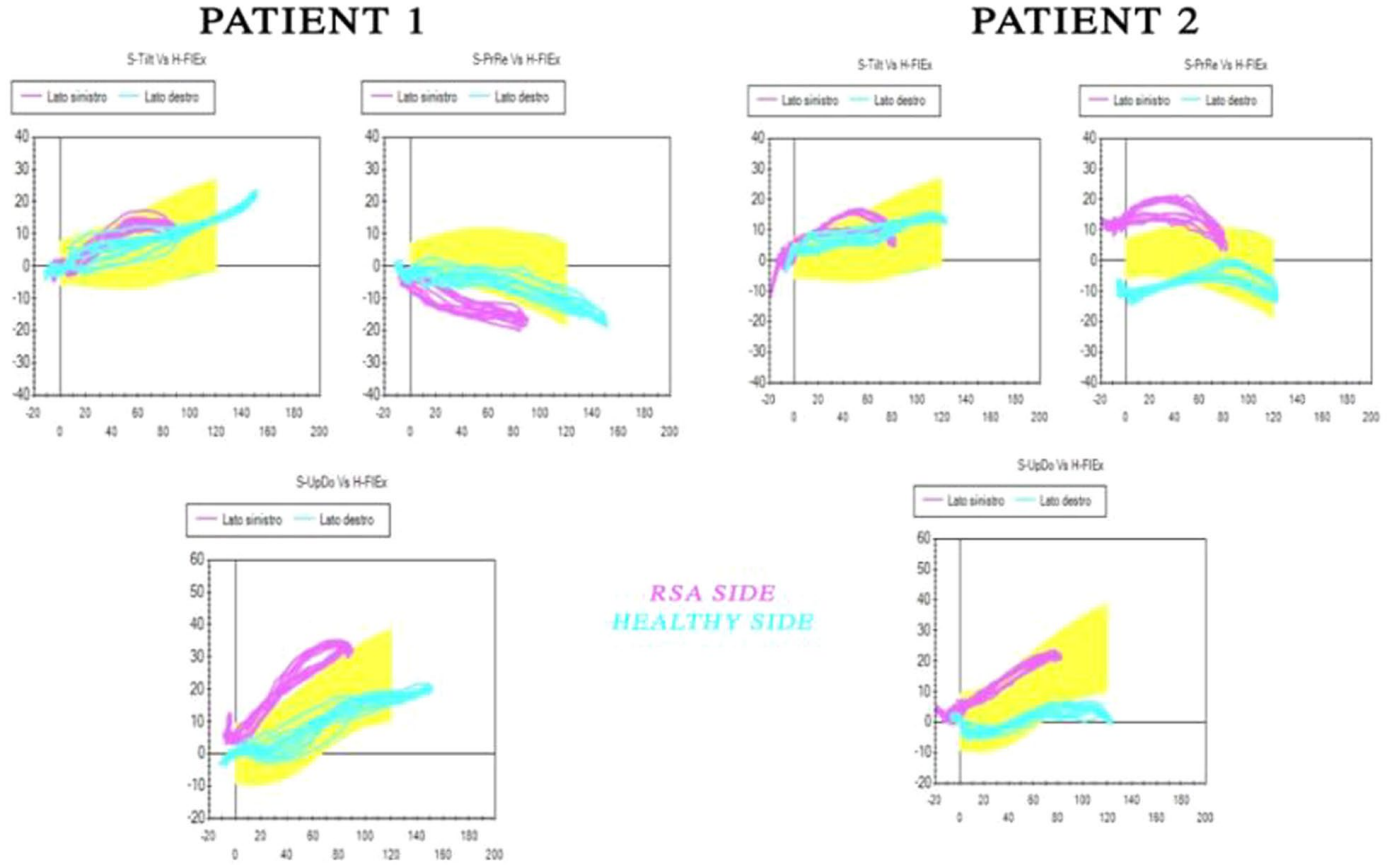
Shoulder Motion analysis output from the system used in the study. A set of six graphs that pit the variations of the three scapular angles studied (anterior/posterior tilt [S-Tilt]) upward/downward rotation [S-UpDo], internal/external rotation [S-PrRe] during Flexion motion (H-Flex) of shoulder comparing healthy and RSA side ( blu and pink lines respectively). Similar morphology of RSA lines can be seen in the graphs between two different OBPI patients.

Post-surgical evaluations of the patients included in the present study with shoulder motion analysis documented a loss of the expected scapular tilting after 80 degrees of active flexion and abduction (Fig. 5), shifting to a major scapular retraction pattern to compensate ROM. Other findings were the loss of external rotation on every position of the arm due to the marked hypotrophy of posterior humeral muscles.

## Discussion

Our cohort of patients had an acceptable clinical outcome at 2 years of follow-up, resulting that RSA being a good solution for Glenohumeral arthritis limitation in OBPI patients. No complications requiring further surgery occured. Considering the preoperative low functioning clinical profile, the patients appeared extremely satisfied with the remarkable reduction in perceived pain and a partial increase in range of motion; pain before surgery was reported mainly at night, with a slow progressive worsening over the years.

In our experience, the results of RSA in OBPI patients showed that even if patients are satisfied after surgery, ROMs are not optimal for the aforementioned limitations in internal rotation and maximum elevation because of the subverted anatomy of these shoulders, also resulting in a specific scapular rhythm pattern.

Comparing RSA in OBPI patients to patients not affected by OBPI subjected to the same type of surgery some differences can be found in this study. First of all, because of the marked hypotrophy especially of the external rotator muscles and albeit minor of the abductor muscles, OBPI patients showed some difficulty in maintaining suspension of the upper limb. Secondly, we can see a decreased external rotation, showing an internal rotation attitude during the movement and an increase in the scapulothoracic motion.

Scapulothoracic movement after RSA surgery has a predominant task in achieving a proper full movement of the shoulder.

It has been demonstrated [14] that in RSA, a significant scapular contribution in posterior tilting, retraction, and anticipated extra-rotation already at 30 degrees of flexion and abduction must be encouraged to help the deltoid, as a motor of the humeral phase, to obtain a favorable lever arm.

**Fig. 6:**
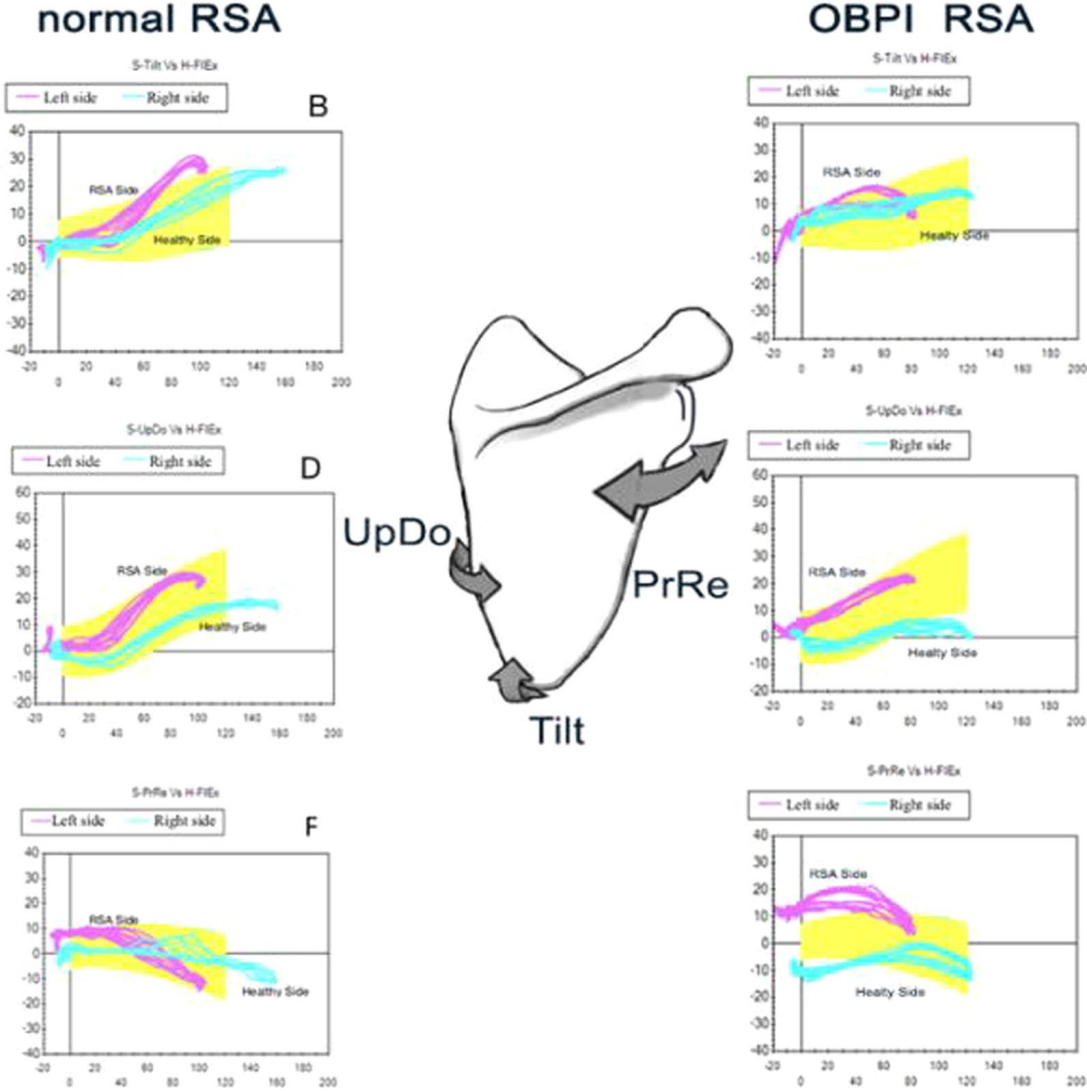
Shoulder Motion analysis RSA in the comparison pattern between a “normal” and an OBPI shoulder. Graphs recorded during Flexion motion show the main specific differences in Tilting and Retraction of the Scapula.

Shoulder Motion analysis of RSA in OBPI showed a complete loss of the scapular tilting above 80 degrees of flexion and abduction compared to the analysis performed on RSA in patients not affected by OBPI in literature [14], thus because they probably miss the top part of elevation ed external rotation for hypotrophy of the posterior deltoid. Our results showed a pattern shifted to the scapular retraction (engaging trapezius and rhomboid muscles) to compensate for the loss of the posterior tilting of the scapula (Fig. 6).

These patients are unable to properly extrapolate and to conclude the humeral phase during the elevation of the arm as happens in normal RSA, therefore in the last degrees the lever on the scapula remains longer and, as a consequence, the tilting flattens, or even it reverses, and as compensation the retraction increases.

Few studies in the literature [4, 7, 16] described the results of total shoulder arthroplasty in patients with Erb palsy, none focused on RSA and analysis of the movement of the scapula.

Werthel et al. [4] focused on the unbalanced force couple in OBPI shoulders, resulting in the inability of the humeral head to maintain the stability required for overhead function with the weakness of external rotators.

Rudge et al. described the results after linked Total shoulder arthroplasty in OBPI patients, showing smaller improvement in function and high complication rates.

This work shows encouraging results in properly managing the arthritic evolution of the shoulder in OBPI patients.

## Limitations

The first limitation is represented by the small sample size of patient. Subsequent analyses with more samples and longer follow-ups are necessary for long-term monitoring of RSA in OBPI patients.

The scapulothoracic analysis data used in this study represent only a visual comparison of motion, With our small cohort of patients, it can’t be standardized the motion patterns of an OBPI RSA.

## Conclusions

In light of the results obtained, RSA is a feasible option for patients affected by OBPI, proving to be very effective in pain control and quality of life though not as good in improving all shoulder motions like external rotation and maximum flexion due to the posterior shoulder muscle hypotrophy that in our experience led to a consequent major retraction of scapulothoracic motion against the physiological posterior scapular tilt of standard RSA.

## Data Availability

The datasets used during the current study are available from the corresponding author on reasonable request.

## Abbreviations

OBPI: Obstetric brachial plexus injury
RSA: Reverse total shoulder arthroplasty
3D-CT: 3D Computer tomography scan
3D-CT: 3D Computer tomography scan
MRI: Magnetic resonance imaging
VAS: Visual analog scale
ROM: Range of motion
BIO-RSA: Bony increased offset- reverse shoulder arthroplasty
OSS: Oxford shoulder score
